# Effects of DNA preservation solution and DNA extraction methods on microbial community profiling of soil

**DOI:** 10.1007/s12223-021-00866-0

**Published:** 2021-04-09

**Authors:** Paul Iturbe-Espinoza, Bernd W. Brandt, Martin Braster, Matthijs Bonte, David M. Brown, Rob J. M. van Spanning

**Affiliations:** 1 Systems Biology Lab, Department of Molecular Cell Biology, Vrije Universiteit Amsterdam, De Boelelaan 1108, 1081 HZ Amsterdam, The Netherlands; 2 Department of Preventive Dentistry, Academic Centre for Dentistry Amsterdam (ACTA), University of Amsterdam and Vrije Universiteit Amsterdam, Amsterdam, The Netherlands; 3 Shell Global Solutions International BV, The Hague, The Netherlands; 4 Ricardo Energy and Environment, Harwell, UK

## Abstract

Microbial community profiling using high-throughput sequencing relies in part on the preservation of the DNA and the effectiveness of the DNA extraction method. This study aimed at understanding to what extent these parameters affect the profiling. We obtained samples treated with and without a preservation solution. Also, we compared DNA extraction kits from Qiagen and Zymo-Research. The types of samples were defined strains, both as single species and mixtures, as well as undefined indigenous microbial communities from soil. We show that the use of a preservation solution resulted in substantial changes in the 16S rRNA gene profiles either due to an overrepresentation of Gram-positive bacteria or to an underrepresentation of Gram-negative bacteria. In addition, 16S rRNA gene profiles were substantially different depending on the type of kit that was used for extraction. The kit from Zymo extracted DNA from different types of bacteria in roughly equal amounts. In contrast, the kit from Qiagen preferentially extracted DNA from Gram-negative bacteria while DNA from Gram-positive bacteria was extracted less effectively. These differences in kit performance strongly influenced the interpretation of our microbial ecology studies.

**Supplementary Information:**

The online version contains supplementary material available at 10.1007/s12223-021-00866-0.

## Introduction

In recent years, high-throughput sequencing, such as the 16S rRNA gene amplicon sequencing, has contributed to a detailed understanding of the composition of microbial communities (Sinclair et al. 2015). The sequential steps in this procedure are (i) sampling and DNA extraction, (ii) PCR amplification, (iii) library preparation and sequencing, and (iv) downstream bioinformatic analysis. During the first critical step, DNA extraction should be equally efficient for all bacterial species of the microbial community to relate community structure to phenotypic function (Zoetendal et al. 2001). However, the community profile can be biased if cells are not lysed or DNA is broken down prior to extraction or incompletely purified during extraction.

To minimize bias in the quantification of microbial communities due to the breakdown of cells or DNA, it is recommended to extract the DNA immediately upon soil collection. If this is not possible, preserving the soil samples may be achieved via snap freezing in liquid nitrogen or storing at − 80 °C though these techniques are not always possible in remote areas (Sessitsch et al. 2002; Straube and Juen 2013; Weißbecker et al. 2017). A second alternative is the addition of alcohol (Harry et al. 2000). However, diluted ethanol (between 60 and 80%) can destroy cells, while absolute ethanol promotes fixation of organic compounds, such as humic acids, to nucleic acids (Rissanen et al. 2010). A third alternative is the use of a soil preservation solution that stabilizes the microbial community DNA, even at ambient temperatures. Those preservative agents usually prevent DNase and RNase activity (Tatangelo et al. 2014; Rippin et al. 2016). Based on the handbook of LifeGuard® Soil Preservation, the preservative does not destroy the cells, but protects the viability of bacteria in soil while keeping them in stasis. The effects of these solutions on the DNA extraction efficiency, however, are not currently well understood, nor is it understood whether the agents are consumed by specialized bacteria at ambient temperature. It is not known where these solutions are based on as their ingredients are kept secret by the companies.

An additional difficulty in extracting DNA from the bacteria in soils is the presence of humic acids, which may be co-extracted and interfere with the subsequent molecular manipulations (Lakay et al. 2007). One way to prevent this is the addition of aluminum sulfate to precipitate them before cell lysis (Persoh et al. 2008). Also, polysaccharides and urea have effects similar to humic acids. Purification of these substances to obtain high-quality DNA is laborious and time-consuming (Jackson et al. 1997). Classical DNA isolation methods, such as phenol–chloroform treatments, cannot efficiently remove humic acids (Tsai and Olson 1992; Moreira 1998; Lakay et al. 2007; Lim et al. 2017). DNA extraction kits developed by several molecular biology companies have a much better performance in the removal of humic acids and other types of PCR inhibitors, but their components are trade secrets (Zielińska et al. 2017). It has been revealed that different types of DNA extraction kits show different community compositions and biodiversity indices (Martin-Laurent et al. 2001; Wüst et al. 2016; Zielińska et al. 2017; Soliman et al. 2017; Xie et al. 2018). However, to our current understanding, the impact of the DNA extraction kit on single species and standardized microbial communities from soils has never been studied in detail. Such a study allows a more fundamental view on the effects of DNA preservation and extraction on different types of species and strongly improves the interpretation of microbial community profiles from different ecosystems.

The objectives in this study were (i) to get insight into biases in the microbial community structure associated with the use of DNA preservation solution LifeGuard® (Qiagen, Benelux BV) as compared to adding no preservative, (ii) to determine if bacterial species can use the stabilizing agents in the solution as carbon and free energy source for growth, and (iii) to compare two different types of DNA extraction kit to evaluate their effectiveness. Although many DNA extraction kits are available in the market, we focused on two DNA extraction kits: the DNeasy PowerSoil Kit (Qiagen, Benelux BV; previously the PowerSoil DNA isolation kit from Mo Bio laboratories), and the ZymoBIOMICS™ DNA Miniprep Kit (Zymo Research, Irvine, CA, USA). Our results showed that both the use of the preservation solution and the type of extraction kit culminated in differences in bacterial community profiles.

## Materials and methods

### Soils samples and preservation during transport

The Shell Petroleum Development Company (SPDC) of Nigeria Ltd provided soil samples that were part of a mesocosm landfarming experiment performed in the Niger Delta region of Nigeria and were described by Brown et al. (2017). Soil samples were selected from four mesocosm containers: an unamended control soil with neither oil nor fertilizer (soil only), and three different soils spiked with oil and fertilizer combined with different treatments: no till, 5% w/w kenaf, and 0.5 g/kg rhamnolipid. No till means that the soil at this condition was not tilled during the landfarming experiment. Kenaf is a bulking agent used to increase soil porosity. Samples from these four containers were taken 24 and 110 days after the start of this landfarming experiment. Those collected after 110 days were taken in triplicate, two of them treated with the DNA preservation solution 1:1 (v/w soil) according to the manufacturer’s protocol (LifeGuard® (Qiagen, Benelux BV)) and one of them untreated. Soil samples collected after 24 days were not treated with DNA preservation solution. We used samples at day 24 as a control to compare the community succession of them after 110 days with and without preservation solution. All samples were kept at around 4 °C in a cold storage box with dry ice during 2 days of transportation from Nigeria to The Netherlands. DNA from these samples was extracted with the Qiagen DNA extraction kit for community profiling on the Illumina platform. In the laboratory, a random soil sample collected after 24 days of landfarming was selected and divided in two portions. DNA of each portion was extracted using either the Qiagen or Zymo DNA extraction kits for further 16S rRNA gene amplicon sequencing analysis to compare the resulting community profiles.

### Identification of bacterial strains

*Paracoccus denitrificans* Pd1222 (de Vries et al. 1989)*,* introduced to this study as a representative of Gram-negative species, was provided by the Vrije Universiteit Amsterdam, Faculty of Sciences, Department of Molecular Cell Biology. The other strains used in this study were isolated from Nigerian soils that made part of the landfarming experiment described above. We performed serial dilutions of extracts from the Nigerian soils and plated these on nutrient agar (NB; Nutrient Broth No. 3; 1 g/L meat extract, 2 g/L yeast extract, 5 g/L peptones, 5 g/L sodium chloride, agar 1.5%, pH 7.4 at 37 °C), after which we picked and isolated the colonies with the most abundant similar colony morphology (Iturbe-Espinoza, unpublished). After culturing the isolated bacteria in NB medium, their full-length 16S rRNA gene sequences were amplified using the bacterial specific primers 8F (CAC GGA TCC AGA CTT TGA T(C/T) (A/C) TGG CTC AG) and 1512R (GTG AAG CTT ACG G(C/T) T AGC TTG TTA CGA CTT) (Weisburg et al. 1991; Felske et al. 1997). The resulting PCR products were sequenced bidirectionally by the Sanger method (1000 nt per read) (Macrogen Europe B.V.). Forward and reverse sequences were merged to obtain the complete gene sequence (around 1500 bp) using the MEGA (v 7) software package. Sequences were compared online to sequences deposited in the nucleotide collection (nr/nt) using megablast (default parameters) on the NCBI BLAST (Basic Local Alignment Search Tool, at www.ncbi.nim.nih.gov) (Zhang et al. 2000). The closest matches of our different isolates were strains of *Pseudomonas aeruginosa* (99.5%; 100%; MN911373.1), *Rhodococcus pedocola* (98.4%; 98%; MG547938.1), *Staphylococcus warneri* (99.1%; 100%; MK414943.1), and *Bacillus drentensis* (99.0%; 99%; KU254657.1), with the percentage of identity, percentage of coverage, and accession number between brackets, respectively. Here, we will tentatively term our isolated strains *P. aeruginosa* R7CO16Bb, *R. pedocola* P7UO26Sw, *S. warneri* I1CO2, and *B. drentensis* I1UO1, respectively. Their 16S rRNA gene sequences have been submitted to GenBank with accession numbers MT949934, MT949938, MT950106, and MT950108, respectively.

### Growth conditions and standard microbial community

To test if the DNA preservation solution is biodegradable, individual isolates were incubated in 50-mL Falcon™ tubes with 10 mL of different concentration of the DNA preservative plus mineral media (Cacl_2_ (0.18 mM); KH_2_PO_4_ (7.34 mM); K_2_HPO_4_ (5.74 mM); NH_4_NO_3_ (12.5 mM); and MgSO_4_ (1.66 mM); pH 6.9, at 30° C and shaken at 200 rpm. The optical density at 600 nm (OD_600_) of the media was measured at 0, 4, 24, 30, 50, 75, and 120 h of incubation to quantify microbial growth.

To design a standard microbial community, the five strains described in the previous section were cultured in 50-mL Falcon™ tubes with 15 mL of Nutrient Broth, overnight at 30 °C and shaken at 120 rpm. The tubes were centrifuged at 3220×*g* for 15 min to spin down the cells, after which the liquid phase was discarded. The pellet was resuspended in phosphate-buffered saline (PBS) at pH 7 (NaCl (0.137 M); KCl (2.7 mM); KH_2_PO_4_ (1.4 mM); Na_2_HPO_4_ (0.01 M) and centrifuged at 3220×*g* for 15 min to wash the cells. The cells were resuspended in PBS to measure the OD_600_. The OD_600_ was adjusted to 1.0. The same volume of the cell suspension of each strain was mixed to generate a standard microbial community in triplicate.

### DNA extraction

To avoid cross-contamination of the samples, the process was performed with DNA free equipment. DNA was extracted with the DNeasy PowerSoil Kit (Qiagen, Benelux BV; previously the PowerSoil DNA isolation kit from Mo Bio laboratories), and the ZymoBIOMICS DNA Miniprep Kit (Zymo Research, Irvine, CA, USA). 300 µL of bacteria cell suspension in PBS in case of single strains or 250 µg of soil were disrupted by shaking in a FastPrep 24 5G (CA, USA) at 6.0 m/s for 60 s. These kits include ready to use tubes with beads. Samples were subsequently processed according to the manufacture’s protocols. The final elution of DNA was 100 µL in all cases. After extraction, DNA concentrations were measured by a Qubit 3.0 fluorometer (Invitrogen, Life technologies) using the Qubit dsDNA HS (high sensitivity) kit (Thermo Fisher Scientific). DNA samples were stored at − 20 °C until further use.

### PCR amplification and sequencing

The DNA concentration in the samples was adjusted to 0.01 ng/µL with RNase/DNase-free water (MP Biomedicals) and distributed in a 96-well plate for PCR amplification of the V3–V4 region of the 16S rRNA gene. Each reaction was performed in a final volume of 25 μL using 10 mM dNTP mix, 2 U/µL Phusion Hot Start II High-Fidelity DNA Polymerase (Thermo Fisher Scientific), 0.1 ng DNA template and 24 primers (8 forward primers; 16 reverse primers) that contains Illumina adapters and an 8-nucleotide index barcode sequence (Kozich et al. 2013). The 16S rRNA gene V3/V4 region of the primers is shown in Supplementary Table 1. The negative PCR control used RNase/DNase-free water as template. To correct for PCR bias, samples were amplified three times, while 35-cycle amplification reactions were performed: 10 s at 98 °C (denaturation), 30 s at 55 °C (annealing), and 30 s at 72 °C (elongation). Triplicate PCRs were first combined, after which PCR products were purified using Agencourt AMPure XP magnetic beads (Beckman Coulter Netherlands B.V.). Next, 40 ng of each product was pooled to get an equimolar mix described earlier (Poursat et al. 2020) for subsequent paired-end sequencing of amplicons on the Illumina MiSeq platform (Illumina, San Diego, USA). The raw sequencing data were deposited in the BioProject database of the National Center for Biotechnology Information (NCBI) under accession number PRJNA660491.

**Table 1:** Fold change in the relative abundance of the five most abundant Gram-positive taxa. The column at the right shows the genus or family level (in cases of unclassified genera). The fold change was calculated from the average of the relative abundance at 110 days of landfarming (with or without preservation solution) compared with 24 days of landfarming

	No till		Rhamnolipid		Kenaf		Soil only	
Taxa	110	110 + PS	110	110 + PS	110	110 + PS	110	110 + PS
*Bacillaceae*	2.6	14.8	1.7	5.4	1.1	2.0	4.6	6.3
*Bacillus*	2.0	4.2	1.9	2.7	2.8	37.0	1.0	3.0
*Lysinibacillus*	2.7	12.8	1.7	26.1	1.5	99.4	0.9	4.6
*Planococcaceae*	5.6	481.3	1.7	22.3	1.6	14.1	1.0	3.0
*Paenibacillus*	2.3	144.3	2.1	138.5	1.9	608.3	1.0	46.9

### Data analysis

Sequencing reads were processed into an OTU table using USEARCH, as previously described (Persoon et al. 2017) with the following differences. After merging, and before clustering, all sequences were additionally quality-filtered using a maximum expected error rate of 0.005, no ambiguous bases allowed. Next, sequences passing the maximum expected error rate of 0.002 were clustered into OTUs using the default sequence similarity threshold of 97%. Finally, the sequences passing the 0.005 error threshold were mapped to the cluster centroids to produce the OTU table. For taxonomic assignments, SILVA v 138.1 (Quast et al. 2013) provided by mothur was used. The SILVA sequences were first trimmed to the V3–V4 16S rRNA gene region and then used to taxonomically classify the representative sequences of the OTUs using mothur v 1.44.3 (default parameters) (Schloss et al. 2009).

The dataset was subsampled to 4850 reads per soil sample for the preservation solution test, 22,300 reads per soil sample for the DNA extraction kit test, and 26,600 reads per standardized microbial community sample. The taxonomic plots are based on relative abundances of the non-subsampled data, after removal of samples not passing the stated subsampling depth. The alpha-diversity indexes, OTU richness (number of OTUs per sample) and Shannon diversity index, were calculated using the subsampled OTU tables. We divided the Shannon index by the binary logarithm of OTU richness to obtain the Pielou’s evenness index (value 0–1) (Pielou 1966). Additionally, a principal coordinate analysis (PCoA) plot was created in R v 3.6.2 (R Development Core Team 2020) via the function plot_ordination (Bray–Curtis distance) of phyloseq v 1.30.0 (McMurdie and Holmes 2013) to compare the beta-diversity and to uncover clustering of soil samples (using the subsampled OTU table). Before generation of bar plots with relative abundances of the genera, those with an abundance of less than 0.1% were removed. Paired *t* tests (Excel) and Wilcoxon signed-rank tests (R) were used to calculate differences in the relative contribution of the most abundant phyla.

## Results

### Effect of the DNA preservation solution on the DNA extraction efficiency

**Fig. 1:**
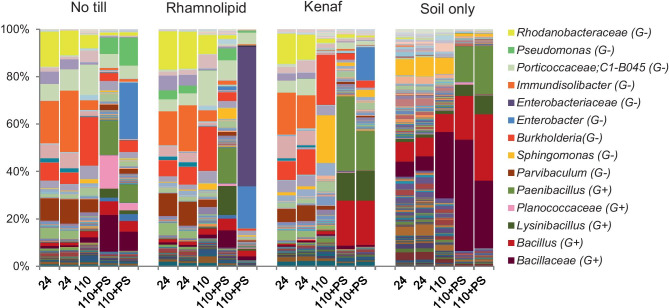
Taxonomic plot at the genus level showing the effect of DNA preservation solution on the soil microbial profiles after 24 and 110 days of landfarming. DNA was extracted using the PowerSoil Kit (Qiagen) of soils from four experimental conditions: only fertilizer no-till, rhamnolipid 0.5 g/kg and fertilizer, 5% w/w kenaf, and fertilizer and soil only (control). Numbers indicate the number of days of landfarming. Samples of 110 days of landfarming were additionally treated with a DNA preservation solution (+ PS). Bar plots of 24 and 110 + PS are biological duplicates. The legend on the right shows the genus or family level (in cases of unclassified genera) with a relative abundance higher than 10% in at least one soil sample. G+, Gram-positive; G−, Gram-negative

**Fig. 2:**
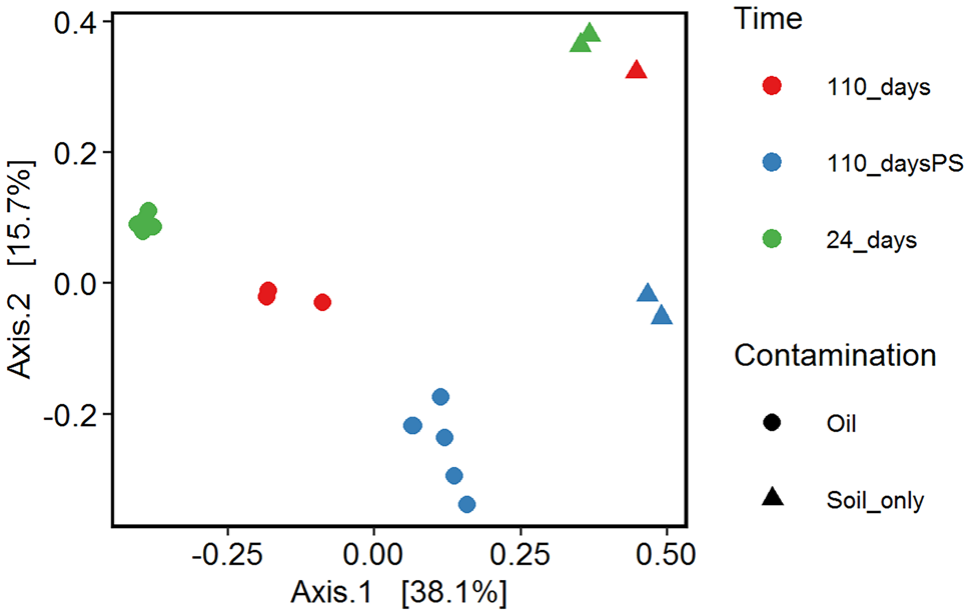
Principal coordinates analysis (PCoA) plot using Bray–Curtis distance. PS: preservation solution (LifeGuard®). Soils were contaminated with crude oil and then biotreated at four conditions: only fertilizer no-till, rhamnolipid 0.5 g/kg and fertilizer, 5% w/w kenaf and fertilizer, and soil only (control)

The effect of the DNA preservation solution on the DNA extraction efficiency was studied with four different soil samples from a previous mesocosm experiment (Brown et al. 2017). The characteristics of these samples are outlined in the section “Materials and methods.” In all four cases where no preservation solution was added to the samples, a clear succession between 24 and 110 days of landfarming is visible, both in the bar plots of relative abundances and in the PCoA analyses (Figs. 1 and 2, respectively). The community profiles of the samples taken at day 24 look much more similar to the untreated samples at day 110 than to those that were treated with the preservation solution. The duplicates of the community profiles of soils after 110 days, which were treated with preservation solution, were not similar. When we compared the corresponding bacterial profiles with those from the untreated samples (soil only), we observed a substantial difference. In all cases, the relative abundances of particularly Gram-positive genera, such as *Bacillus, Paenibacillus, Lysinibacillus*, and unclassified members of the family *Bacillaceae* and *Planococcaceae,* increased for the samples treated with DNA preservation solution as compared to the ones from samples delivered without preservation solution (Fig. 1). Table 1 shows the fold change of the relative abundance of the most dominant Gram-positive bacteria. The relative abundances of the most abundant genera are shown in Supplementary Table 2. The PCoA ordination plot of these samples shows clear differences between the soils and especially high variability in the samples treated with the DNA preservation solution (Fig. 2). This result indicates that DNA preservation solution has a substantial effect on the bacterial profiling resulting in biased community profiles as compared to those not treated with the preservation solution.

**Table 2:** Fold change in the relative abundances of phyla obtained using the Qiagen extraction kit as opposed to those obtained using the kit from Zymo. The list includes phyla with a mean relative abundance higher than 1% in at least one soil sample

Gram staining	Phylum	Fold change	*p* value (paired *t* test)	*p* value (Wilcoxon signed‐rank test)
Gram-negative	Acidobacteriota	1.2	0.288	0.5
	Chloroflexi	0.6	0.120	0.5
	Planctomycetota	0.2	0.165	0.5
	Proteobacteria	1.2	0.167	0.5
	Verrucomicrobiota	1.4	0.057	0.5
Gram-positive	Firmicutes	0.5	0.332	0.5
	Actinobacteriota	0.3	**0.014**	0.5

### Use of agents in the preservation solution as carbon and free energy metabolism for growth

**Fig. 3:**
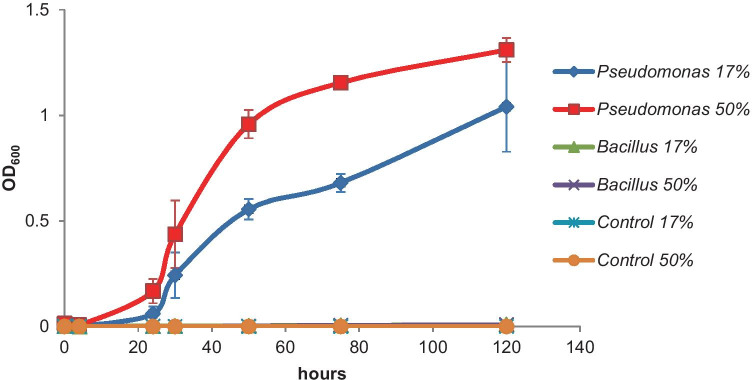
Growth curves of *Pseudomonas aeruginosa R7CO16Bb* and *Bacillus drentensis I1UO1* isolated from Nigerian soils in mineral media with Soil Preservation solution (LifeGuard®) added to it at concentrations of 17% and 50% v/v. The control was a flask without bacterial inoculum. Incubation was at 30 °C, with a shaking frequency of 200 rpm. Data points are the averages of duplicates with the SD values shown

To test if the DNA preservation solution is biodegradable by and growth supporting for bacteria, we incubated *B. drentensis I1UO1* and *P. aeruginosa R7CO16Bb* in mineral media with the DNA preservation solution as sole source of carbon and free energy. *B. drentensis I1UO1* did not grow on it. Here, we can likely rule out that the changes in community profiles that we observed were the result of growth of *Bacillus* species. However, *P. aeruginosa R7CO16Bb* grew in the media with the DNA preservation solution both at concentrations of 17% and 50% (Fig. 3).

### Effect of the DNA extraction kit on the DNA extraction efficiency

#### DNA extraction efficiency using natural microbial communities

**Fig. 4:**
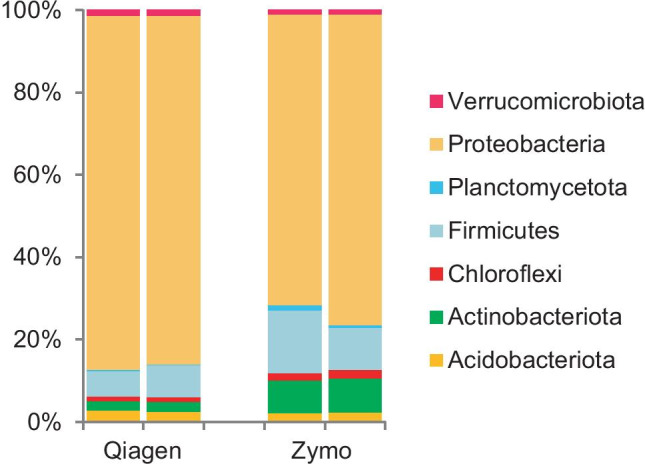
Comparison of the microbial profiles (relative abundances) of a soil microbial community at the phylum level using DNeasy PowerSoil (Qiagen) and ZymoBIOMICS™ (Zymo) DNA extraction kits. Bars are biological duplicates

One of the samples we took to study the effect of the DNA extraction kit on the efficiency of DNA extraction was taken after 24 days of landfarming of the mesocosm container filled with soil, oil, and fertilizer but not tilled. The sample was split into two portions after which we isolated the DNA with either the DNeasy PowerSoil Kit (Qiagen, Benelux BV), or ZymoBIOMICS DNA Miniprep Kit (Zymo Research, Irvine, CA, USA) and subsequently performed 16S amplicon sequencing of both DNA extracts. The results show that the relative abundance of the phyla Actinobacteriota and Firmicutes was significantly higher and that of the Gram-negative species lower in the microbial profiles of the DNA extracts obtained with the Zymo kit as compared with those obtained with the Qiagen kit. Apart from these two Gram-positive phyla, also Planctomycetota and Chloroflexi were relatively more abundant with the Zymo kit as compared with the Qiagen kit. Table 2 shows the fold change between the two approaches and corresponding p-values (paired *t* test and Wilcoxon signed‐rank test based on relative abundances). The *p* values are not significant due to the small sample size (*N* = 4) with the exception of the one of the Gram-positive phylum Actinobacteriota. Obviously, the relative abundances of species from other phyla, like the Proteobacteria, are higher when the DNA extraction kit from Qiagen is used as compared to those using the Zymo kit (Fig. 4).

**Fig. 5:**
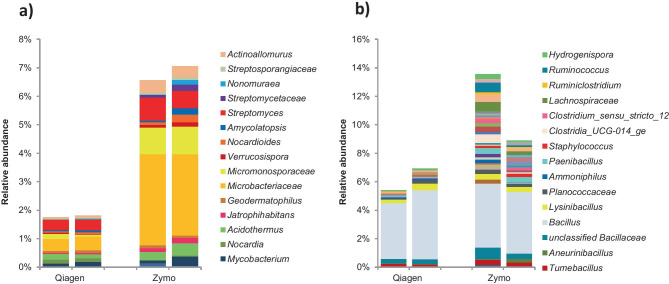
Comparison of relative abundances of microbial profiles from genera in the phyla Actinobacteriota **a)** and Firmicutes **b)** from a soil sample contaminated with crude oil and treated with fertilizer but not tilled. DNA was extracted using the DNeasy PowerSoil kit (Qiagen) or the ZymoBIOMICS™ kit (Zymo). Bars are biological duplicates

More details about the differences in extraction efficiency between the two extraction kits are visualized in Fig. 5, where the relative abundances of the genera of Actinobacteriota and Firmicutes, which are mainly Gram-positive, are shown. The relative abundances of the genera belonging to all phyla in this community are shown in Supplementary Fig. 1. Although the richness indices of the microbial profiles are similar regardless of the type of DNA extraction kit, we observed differences in Shannon and evenness indices (Supplementary Fig. 2). The genera in the Zymo profiles are distributed more equally than those in the Qiagen profiles although the number of samples is too limited (*N* = 4) to do any useful informative statistics.

#### DNA extraction efficiency using single strains

**Fig. 6:**
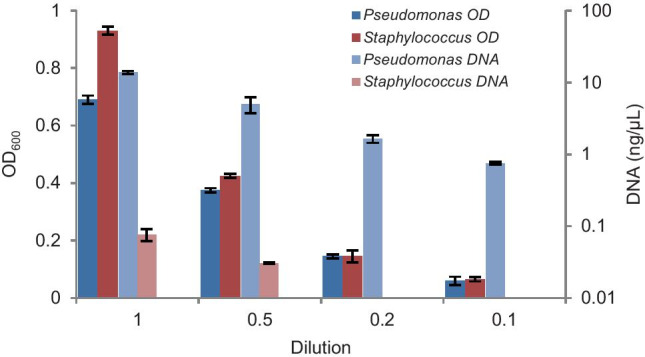
Comparison of DNA extraction efficiency between *Pseudomonas aeruginosa R7CO16Bb* and *Staphylococcus warneri I1CO2* using the DNeasy PowerSoil Kit (Qiagen). Cells were diluted at different optical densities at 600 nm (OD_600_) for DNA extractions. DNA concentration was measured by a Qubit 3.0 fluorometer and expressed in ng/µL. Data points are averages of a duplicate with the SD values shown

We evaluated in more detail what the efficiencies of the Qiagen DNA extraction kit are using cultures from individual species, both a Gram-positive one, *S. warneri I1CO2*, and a Gram-negative one, *P. aeruginosa R7CO16Bb*. We observed significant differences in DNA extraction efficiency between these species at comparable cell densities, as reflected by their OD_600_ values (Fig. 6). DNA extracted from *P. aeruginosa R7CO16Bb* was about 50 times higher than that from *S. warneri I1CO2* at all comparable cell densities tested*.*

**Fig. 7:**
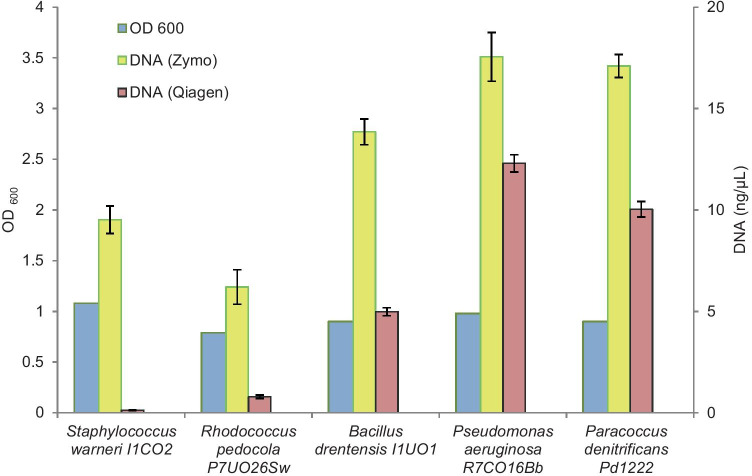
Comparison of efficiencies of the DNeasy PowerSoil Kit (Qiagen) and the from ZymoBIOMICS™ (Zymo) DNA extraction kit for different isolated strains. The OD_600_ of the cell suspensions were adjusted to around 1. DNA extractions were performed in duplicate with the standard deviations as shown

Next, we compared the Zymo and Qiagen DNA extraction kits using cell suspensions of the Gram-positive species *S. warneri I1CO2*, *R. pedocola P7UO26Sw* and *B. drentensis I1UO1*, and the Gram-negative species *P. aeruginosa R7CO16Bb*, and *P. denitrificans Pd1222*, all with comparable biomasses (OD_600_ of around 1). In all cases, the DNA extraction efficiency of the Zymo kit was higher than that of the Qiagen kit (Fig. 7), especially when it concerned the cultures from the Gram-positive species. Most profound is the around 50-fold difference in efficiency for the *S. warneri I1CO2* species in favor of the Zymo DNA extraction kit.

**Fig. 8:**
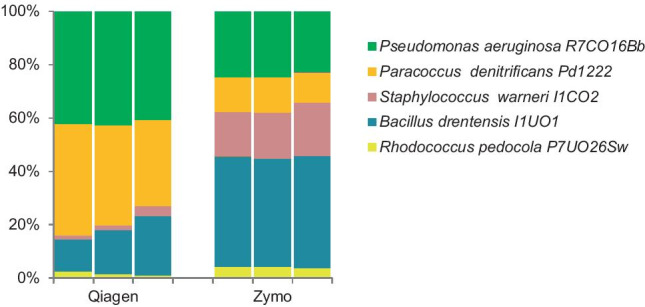
Comparison of relative abundances of five species all with equal contributions in a standardized microbial community on the basis of their microbial profiles after extraction of their DNA using DNeasy PowerSoil Kit (Qiagen) or ZymoBIOMICS™ (Zymo). The three bars are results of technical replicates

This difference is further corroborated by comparing the microbial profiles of a standardized mixture of these five strains, all with similar optical densities (Fig. 8). The profiles obtained using the Zymo DNA extraction kit scored better as judged by the more equal and reproducible distribution of the five species. It should be noted, however, that this result did not take the numbers of 16S rRNA gene copies in the genome of the five strains into account.

## Discussion

We showed that the DNA extraction efficiency is affected by the type of preservation solution to store the sample during transport and by the type of kit used to extract the DNA. Our data show that the use of a preservation solution for soil samples results in higher relative abundances of Gram-positive species versus Gram-negative species as opposed to samples not treated with it. Based on that, we hypothesize that the bias observed may be the result of decreased membrane integrity resulting in enhanced efficiency of DNA extraction from mostly Gram-positive bacteria. Alternatively, the DNA preservation solution may make membranes of Gram-negative species more porous resulting in enhanced release of the DNA and its subsequent breakdown, also leading to increased relative abundances of Gram-positives, such as *Bacillus, Paenibacillus, Lysinibacillus*, and unclassified members of the family *Bacillaceae* and *Planococcaceae* as shown in Table 1. We therefore recommend that the use of preservation solution in future ecological studies on comparable types of sample (i.e., soils) should be critically reviewed. A previous study on soil microbial communities reported that the use of preservation solutions (ethanol and RNAlater®) caused an increase of the nucleic acid yields of the Chloroflexi, Alphaproteobacteria, and Gram-positive Actinobacteriota with a subsequent increased representation of them in the total microbial community profile (Rissanen et al. 2010). It is interesting to note that although this study used a different stabilization agent, a similar positive bias towards Gram-positive bacteria was found. Besides, other studies, albeit focused on feces and aquatic systems, also reported severe changes in the representation of the microbial communities profile using different brands of preservation solutions (McCarthy et al. 2015; Chen et al. 2019). Protecting the DNA in samples is better carried out by other means like cooling or freezing whenever possible. Here, we also take into account the results from studies, which indicate that especially soil samples can be safely stored without a DNA preservation solution at room temperature (Lauber et al. 2010; Tatangelo et al. 2014). This is further supported by our findings that showed that some species, like *P. aeruginosa R7CO16Bb*, have the metabolic capacity to use the DNA preservation solution as a carbon and free energy source at more ambient temperatures. This process may have unwanted effects on the community profiles as well since these degraders may obtain a higher contribution in the community as a result of that when transportation conditions are not fully controlled according to the protocol of the manufacturer. This protocol indicates the soil microbial community profiles are maintained stable with the preservation solution for up to one week at room temperature.

The efficiency of DNA extraction varied both between the two different kits used in our study as well as between single strains, synthetic communities, and communities from soil samples. The use of mock communities is a powerful tool to assess the reliability of microbial community sequence analysis. It shows that these differences mainly concern the extraction of DNA from Gram-positive species, which is carried out much more efficiently by the ZymoBIOMICS™ DNA Miniprep Kit. The extraction of DNA also differs between different Gram-positive species, with *S. warneri I1CO2*,* R. pedocola P7UO26Sw*, and *B. drentensis I1UO1* sorted from difficult to easy to extract. In general, Gram-positive bacteria are difficult to break open due to the thick multilayered and extensively cross-linked peptidoglycan layer in contrast to the thinner layer of peptidoglycan in Gram-negative cells (Mahalanabis et al. 2009). Also, other studies revealed that the use of different DNA extraction kits may result in significantly different community profiles as a consequence of poor performance in DNA extraction from mainly Gram-positive bacteria (Wüst et al. 2016; Zielińska et al. 2017; Xie et al. 2018). However, we also noted a better performance of the Zymo kit for the extraction of DNA from Gram-negative species from the phyla Planctomycetota and Chloroflexi. Planctomycetota have cell-wall sacculi that contain peptidoglycan, the disruption of which is improved by the addition of lysozyme (Pilhofer et al. 2013; Jeske et al. 2015; van Teeseling et al. 2015), indicating that this cell wall is robust and difficult to break open. Chloroflexi are monoderms with their cytoplasmic membrane surrounded by a cell wall of polysaccharides associated to peptidoglycan. In that respect, their cell wall resembles that of Gram-positive bacteria (Sutcliffe 2011; Speirs et al. 2019). The strength of the peptidoglycan layer in the composition of their cell walls may affect the efficiency of DNA extraction as well. The resulting bias has important consequences for many types of ecological study. In the event that the abundance of Gram-positive species, and Planctomycetota and Chloroflexi is underestimated, their potential role in the food web that they make part of is masked, which prevents a more fundamental understanding of the structure, function and dynamics of the community along with all of its interactions. For future microbial community profiling studies, we recommend to test different types of preservation solution and DNA extraction kit and select those materials and methods that show the best performance in terms of accurate microbial community profiling. Prior to submission of this article, we were notified by Qiagen that their DNeasy PowerSoil kit is replaced by the DNeasy Powersoil Pro kit, which improves DNA extraction of Gram-positive bacteria (Sperling et al. 2018).

## Supplementary Information

Below is the link to the electronic supplementary material.

Supplementary file1 (PPTX 76 KB)

Supplementary file2 Supplementary Figure 1 Comparison of relative abundance of microbial profiles at the genus (or higher) level of the microbial community using two DNA extraction kits: DNeasy PowerSoil Kit (Qiagen) and ZymoBIOMICS™ (Zymo). Only the most abundant genera (the top 17) are listed in the legend. The genera are grouped in bacterial phyla and subphyla (in the case of Proteobacteria). (PPTX 52 KB)

Supplementary file3 Supplementary Figure 2 Alpha-diversity indices. DNA was extracted using two DNA extraction kits DNeasy PowerSoil Kit (Qiagen) and ZymoBIOMICS™ (Zymo). Standard deviations were calculated from two biological replicates. a) Richness represents the total number of OTUs per sample. b) Shannon represents the relative abundance of each OTU. c) Pielou’s evenness represents the evenness of OTUs abundances within the community profile. (DOCX 17 KB)

## Data Availability

All data generated or analyzed during this study are included in this article or at the public database of NCBI with accession numbers presented.
