# Supplementary material for: Effects of DNA preservation solution and DNA extraction methods on microbial community profiling of soil
Source: Folia Microbiol (Praha). 2021 Apr 9;66(4):597–606. doi: 10.1007/s12223-021-00866-0 (PMC8298342; doi:10.1007/s12223-021-00866-0)
Supplement: Supplementary file 1 — Supplementary file1 (PPTX 76 KB) [file 12223_2021_866_MOESM1_ESM.pptx]

## Slide 1
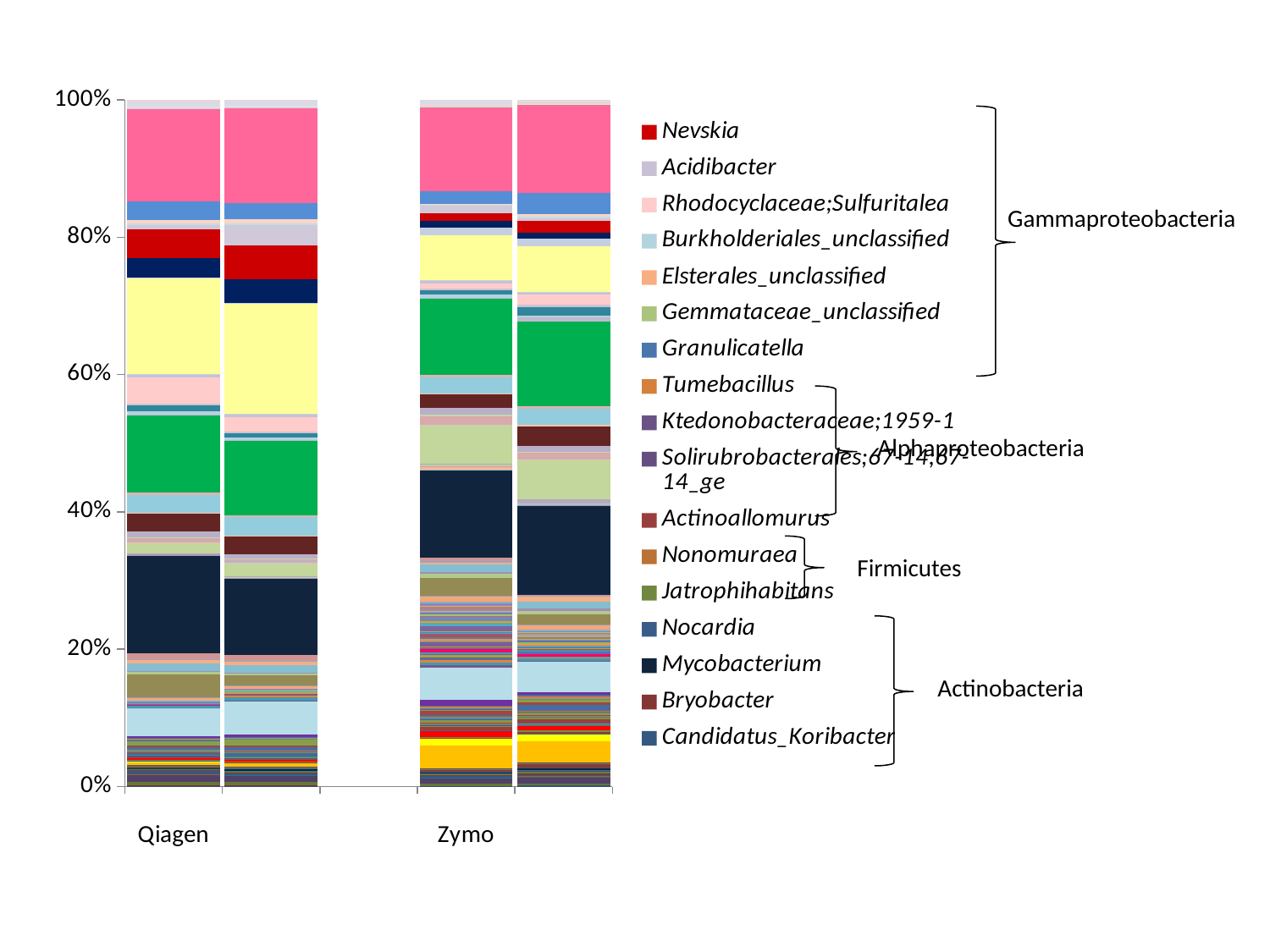

### Chart
| Category | Methanobacterium | Paludibaculum | Acidipila-Silvibacterium | Acidobacteriaceae_(Subgroup_1)_unclassified | Terriglobus | Acidobacteriales_unclassified | Candidatus_Koribacter | Bryobacter | Candidatus_Solibacter | uncultured | Actinobacteria_unclassified | Mycobacterium | Nocardia | Acidothermus | Jatrophihabitans | Geodermatophilus | Microbacteriaceae | Micromonosporaceae | Micromonosporaceae;Verrucosispora | Nocardioidaceae;Nocardioides | Pseudonocardiaceae;Amycolatopsis | Streptomyces | Streptomycetaceae_unclassified | Nonomuraea | Streptosporangiaceae_unclassified | Actinoallomurus | uncultured;uncultured_ge | Solirubrobacterales;67-14;67-14_ge | Conexibacter | Bacteroides | Chitinophagaceae;uncultured | Oligoflexia;0319-6G20;0319-6G20_fa;0319-6G20_ge | JG30-KF-AS9;JG30-KF-AS9_ge | Ktedonobacteraceae;1959-1 | Ktedonobacteraceae;FCPS473 | Ktedonobacteraceae;HSB_OF53-F07 | Ktedonobacteraceae_unclassified | Ktedonobacteraceae;uncultured | Obscuribacteraceae_ge | Babeliales_unclassified | Alicyclobacillus | Tumebacillus | Aneurinibacillus | Bacillaceae_unclassified | Bacillus | Geobacillus | Lysinibacillus | Planococcaceae_unclassified | Granulicatella | Streptococcus | Ammoniphilus | Cohnella | Paenibacillaceae_unclassified | Paenibacillus | Staphylococcus | Christensenellaceae_R-7_group | Christensenellaceae;uncultured | Clostridia_UCG-014_ge | Clostridia_unclassified | Clostridium_sensu_stricto_1 | Clostridium_sensu_stricto_10 | Clostridium_sensu_stricto_12 | Clostridium_sensu_stricto_3 | Clostridium_sensu_stricto_8 | Blautia | Coprococcus | Lachnospiraceae_ND3007_group | Lachnospiraceae_NK4A136_group | Lachnospiraceae_ge | Lachnospiraceae_unclassified | Hungateiclostridiaceae_unclassified | Ruminiclostridium | Ruminococcus | Subdoligranulum | Firmicutes_unclassified | Hydrogenispora | Anaeromyxobacter | Haliangium | Pajaroellobacter | WD2101_soil_group_ge | Gemmataceae_unclassified | Gemmataceae;uncultured | Isosphaeraceae_unclassified | Acetobacteraceae_unclassified | Acetobacteraceae;Acidisphaera | Acidocella | Acetobacteraceae;uncultured | Defluviicoccus | Dongia | Elsterales_unclassified | Elsterales;uncultured;uncultured_ge | Micropepsaceae;uncultured | Parvibaculum | Amb-16S-1323;Amb-16S-1323_ge | Beijerinckiaceae_unclassified | Methylobacterium-Methylorubrum | Methylocella | Methylocystis | Hyphomicrobium | Pedomicrobium | Bradyrhizobium | Xanthobacteraceae_unclassified | Xanthobacteraceae;uncultured | Sandaracinobacter | Sphingobium | Sphingomonadaceae_unclassified | Sphingomonas | uncultured_ge | Acidithiobacillaceae;KCM-B-112 | Alcaligenaceae;Bordetella | Burkholderia | Ralstonia | Burkholderiales_unclassified | Comamonadaceae_unclassified | Comamonadaceae;Rhizobacter | Oxalobacteraceae_unclassified | Rhodocyclaceae;Sulfuritalea | Acidibacter | Gammaproteobacteria_unclassified | Immundisolibacter | Legionella | Porticoccaceae;C1-B045 | Nevskia | Solimonas | Dyella | Luteibacter | Rhodanobacter | Rhodanobacteraceae | Protochlamydia | Parachlamydiaceae_unclassified | Candidatus_Udaeobacter | Chthoniobacter | Methylacidiphilaceae;uncultured | Pedosphaeraceae_unclassified |
|---|---|---|---|---|---|---|---|---|---|---|---|---|---|---|---|---|---|---|---|---|---|---|---|---|---|---|---|---|---|---|---|---|---|---|---|---|---|---|---|---|---|---|---|---|---|---|---|---|---|---|---|---|---|---|---|---|---|---|---|---|---|---|---|---|---|---|---|---|---|---|---|---|---|---|---|---|---|---|---|---|---|---|---|---|---|---|---|---|---|---|---|---|---|---|---|---|---|---|---|---|---|---|---|---|---|---|---|---|---|---|---|---|---|---|---|---|---|---|---|---|---|---|---|---|---|---|---|---|---|---|---|---|---|---|
| Qiagen | 0.000541446435478 | 0.00116024236174 | 0.00495036741008 | 0.00923037256671 | 0.00110867603455 | 0.000257831635942 | 0.0044347041382 | 0.00118602552533 | 0.00170168879722 | 0.0 | 2.57831635942e-05 | 0.0013407245069 | 0.00136650767049 | 0.00198530359675 | 0.00030939796313 | 0.000721928580637 | 0.00422843882944 | 0.00172747196081 | 0.000412530617507 | 0.000567229599072 | 0.000438313781101 | 0.0035322934124 | 5.15663271883e-05 | 0.0 | 0.000335181126724 | 0.000489880108289 | 0.0013407245069 | 0.000902410725796 | 0.000206265308753 | 0.0 | 0.00379012504834 | 0.000128915817971 | 0.000979760216579 | 0.00136650767049 | 5.15663271883e-05 | 0.0013149413433 | 0.00384169137553 | 0.0021915689055 | 0.00742555111512 | 0.000154698981565 | 0.000257831635942 | 0.0022173520691 | 0.000438313781101 | 0.00306819646771 | 0.0389583601908 | 2.57831635942e-05 | 0.00250096686863 | 0.000850844398608 | 0.0 | 0.0 | 0.000335181126724 | 7.73494907825e-05 | 0.000412530617507 | 0.00136650767049 | 0.0 | 0.000103132654377 | 0.0 | 0.0 | 0.000103132654377 | 0.000283614799536 | 0.000412530617507 | 0.000257831635942 | 7.73494907825e-05 | 0.00030939796313 | 0.0 | 0.0 | 0.0 | 0.0 | 0.0 | 2.57831635942e-05 | 0.000644579089854 | 0.000335181126724 | 0.0 | 0.0 | 0.000257831635942 | 0.000721928580637 | 0.000206265308753 | 0.000283614799536 | 2.57831635942e-05 | 0.000850844398608 | 0.000103132654377 | 0.000644579089854 | 0.000232048472348 | 0.00314554595849 | 0.00149542348846 | 0.0324610029651 | 0.00301663014052 | 0.00136650767049 | 0.010416398092 | 0.00476988526492 | 0.00116024236174 | 0.00858579347686 | 0.138146190538 | 0.00286193115895 | 7.73494907825e-05 | 2.57831635942e-05 | 0.0 | 0.000541446435478 | 0.000103132654377 | 0.000180482145159 | 0.0145159211035 | 0.000773494907825 | 0.00108289287096 | 0.00525976537321 | 0.000979760216579 | 0.00786386489622 | 0.0260925615573 | 0.000257831635942 | 0.0262214773753 | 0.00296506381333 | 0.109965192729 | 0.000257831635942 | 0.00466675261055 | 0.00830217867732 | 0.00172747196081 | 0.00139229083409 | 0.036405826995 | 0.00420265566585 | 0.000670362253448 | 0.136599200722 | 0.000696145417043 | 0.0270207554467 | 0.0412272785871 | 0.00611060977182 | 0.0022173520691 | 0.00407373984788 | 0.0266597911564 | 0.131623050148 | 0.00092819388939 | 5.15663271883e-05 | 0.00105710970736 | 0.00116024236174 | 0.00613639293541 | 0.00319711228568 |
| | 0.0012847277983 | 0.000995664043681 | 0.00478561104866 | 0.00822225790911 | 0.000706600289064 | 0.0 | 0.00298699213104 | 0.000674482094106 | 0.00237674642685 | 0.0 | 0.000321181949574 | 0.001509555163 | 0.0012847277983 | 0.00144531877308 | 0.000513891119319 | 0.000899309458808 | 0.00478561104866 | 0.000674482094106 | 3.21181949574e-05 | 0.000835073068894 | 0.000738718484021 | 0.00366147422515 | 3.21181949574e-05 | 0.000321181949574 | 0.00025694555966 | 0.000867191263851 | 0.000867191263851 | 0.000931427653766 | 0.000449654729404 | 0.0 | 0.00507467480328 | 0.0 | 0.000642363899149 | 0.00179861891762 | 0.00025694555966 | 0.000610245704191 | 0.00481772924362 | 0.00247310101172 | 0.00841496707885 | 0.000770836678979 | 0.00025694555966 | 0.0017343825277 | 0.000353300144532 | 0.00334029227557 | 0.0483700016059 | 3.21181949574e-05 | 0.00472137465874 | 0.00228039184198 | 0.0 | 0.0 | 0.000835073068894 | 0.000321181949574 | 0.000578127509234 | 0.000706600289064 | 6.42363899149e-05 | 0.000224827364702 | 0.0 | 0.0 | 0.000192709169745 | 0.000481772924362 | 0.000738718484021 | 0.000481772924362 | 0.00012847277983 | 0.000674482094106 | 0.0 | 0.0 | 0.0 | 0.0 | 0.0 | 0.00012847277983 | 0.000674482094106 | 3.21181949574e-05 | 0.0 | 0.0 | 0.000770836678979 | 0.00115625501847 | 9.63545848723e-05 | 0.00025694555966 | 0.000192709169745 | 0.000289063754617 | 0.000192709169745 | 0.000674482094106 | 0.0 | 0.00404689256464 | 0.000578127509234 | 0.0142283603661 | 0.00250521920668 | 0.00115625501847 | 0.0106953589208 | 0.00433595631925 | 0.0012847277983 | 0.00819013971415 | 0.107852898667 | 0.00176650072266 | 6.42363899149e-05 | 0.0 | 0.0 | 0.000706600289064 | 9.63545848723e-05 | 0.000160590974787 | 0.0193351533644 | 0.000610245704191 | 0.00167014613779 | 0.00266581018147 | 0.00109201862855 | 0.00664846635619 | 0.0257909105508 | 0.000449654729404 | 0.0261442106954 | 0.00183073711257 | 0.1057330978 | 0.00115625501847 | 0.0025694555966 | 0.00757989400996 | 0.000192709169745 | 0.000642363899149 | 0.0204592901879 | 0.00417536534447 | 0.000513891119319 | 0.155130881644 | 0.00170226433274 | 0.0337241047053 | 0.046796210053 | 0.0297735667256 | 0.00231251003694 | 0.00504255660832 | 0.0225469728601 | 0.134607355067 | 0.000802954873936 | 0.000192709169745 | 0.000674482094106 | 0.00131684599326 | 0.00600610245704 | 0.00253733740164 |
| | None | None | None | None | None | None | None | None | None | None | None | None | None | None | None | None | None | None | None | None | None | None | None | None | None | None | None | None | None | None | None | None | None | None | None | None | None | None | None | None | None | None | None | None | None | None | None | None | None | None | None | None | None | None | None | None | None | None | None | None | None | None | None | None | None | None | None | None | None | None | None | None | None | None | None | None | None | None | None | None | None | None | None | None | None | None | None | None | None | None | None | None | None | None | None | None | None | None | None | None | None | None | None | None | None | None | None | None | None | None | None | None | None | None | None | None | None | None | None | None | None | None | None | None | None | None | None | None | None | None | None | None | None | None |
| Zymo | 7.48418964937e-05 | 0.0 | 0.00445309284137 | 0.00752161059761 | 0.000449051378962 | 0.0 | 0.00205815215358 | 0.00183362646409 | 0.00101036560266 | 0.0 | 0.00138457508513 | 0.000972944654418 | 0.000299367585975 | 0.00273172922202 | 0.00123489129215 | 0.0011226284474 | 0.0319574898028 | 0.00931781611346 | 0.000898102757924 | 0.000972944654418 | 0.000598735171949 | 0.00800808292482 | 0.00101036560266 | 0.000149683792987 | 0.000598735171949 | 0.00437825094488 | 0.00142199603338 | 0.000860681809677 | 0.00104778655091 | 0.00172136361935 | 3.74209482468e-05 | 0.00108520749916 | 0.00355499008345 | 0.000523893275456 | 0.00246978258429 | 0.0011226284474 | 0.00288141301501 | 0.00576282603001 | 0.00168394267111 | 0.00011226284474 | 0.00153425887812 | 0.00362983197994 | 0.00071099801669 | 0.00793324102833 | 0.0449799797927 | 0.00276915017027 | 0.00392919956592 | 0.00321820154923 | 0.00333046439397 | 0.00101036560266 | 0.00261946637728 | 0.00168394267111 | 0.0023200987913 | 0.00404146241066 | 0.00145941698163 | 0.00108520749916 | 0.00108520749916 | 0.00617445646073 | 0.00142199603338 | 0.00374209482468 | 0.00246978258429 | 0.00318078060098 | 0.0 | 0.00101036560266 | 0.00127231224039 | 0.0 | 0.0011974703439 | 0.00116004939565 | 0.000374209482468 | 0.00677319163268 | 0.00512666990982 | 0.00153425887812 | 0.00692287542566 | 0.000224525689481 | 0.00235751973955 | 0.00314335965273 | 0.0023200987913 | 0.00011226284474 | 0.00134715413689 | 0.0035924110317 | 0.00145941698163 | 0.00381693672118 | 0.00160910077461 | 0.00722224301164 | 0.00127231224039 | 0.0256707704973 | 0.00550087939228 | 0.00228267784306 | 0.0108146540433 | 0.00246978258429 | 0.00160910077461 | 0.00508924896157 | 0.12188002844 | 0.000748418964937 | 0.000449051378962 | 0.00318078060098 | 0.0 | 0.00116004939565 | 0.00187104741234 | 0.00187104741234 | 0.0522396437526 | 0.00142199603338 | 0.00149683792987 | 0.0104778655091 | 0.00209557310182 | 0.00939265800995 | 0.0194588930884 | 0.000636156120196 | 0.0228641993788 | 0.0029562549115 | 0.10736070052 | 3.74209482468e-05 | 0.00493956516858 | 0.0065486659432 | 0.00145941698163 | 0.000149683792987 | 0.00752161059761 | 0.00310593870449 | 0.00149683792987 | 0.0618194065038 | 0.0116379149048 | 0.00883134378625 | 0.0105901283539 | 0.0102159188714 | 0.00127231224039 | 0.00217041499832 | 0.0172136361935 | 0.117688882236 | 0.000598735171949 | 0.000598735171949 | 0.0 | 0.00108520749916 | 0.00651124499495 | 0.00082326086143 |
Gammaproteobacteria
Alphaproteobacteria
Firmicutes
Actinobacteria
