# Supplementary material for: Effects of DNA preservation solution and DNA extraction methods on microbial community profiling of soil
Source: Folia Microbiol (Praha). 2021 Apr 9;66(4):597–606. doi: 10.1007/s12223-021-00866-0 (PMC8298342; doi:10.1007/s12223-021-00866-0)
Supplement: Supplementary file 2 — Supplementary file2 Supplementary Figure 1 Comparison of relative abundance of microbial profiles at the genus (or higher) level of the microbial community using two DNA extraction kits: DNeasy PowerSoil Kit (Qiagen) and ZymoBIOMICS™ (Zymo). Only the most abundant genera (the top 17) are listed in the legend. The genera are grouped in bacterial phyla and subphyla (in the case of Proteobacteria). (PPTX 52 KB) [file 12223_2021_866_MOESM2_ESM.pptx]

## Slide 1
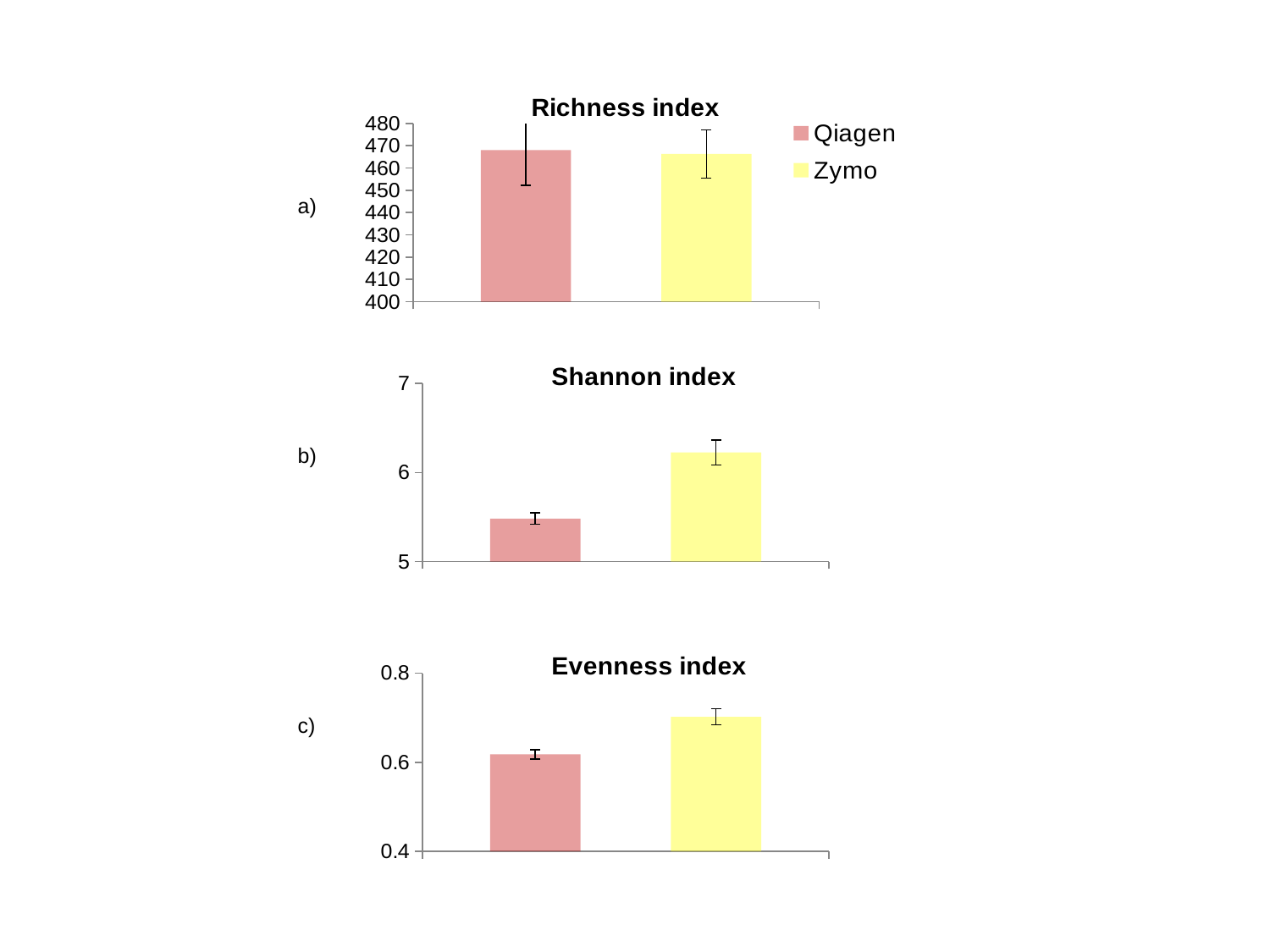

### Chart: Richness index
| Category | Qiagen | | Zymo |
|---|---|---|---|
| Richness | 468.0 | None | 466.35 |a)
### Chart: Shannon index
| Category | Qiagen | | Zymo |
|---|---|---|---|
| shannon Ave. | 5.483499999999999 | None | 6.2255 |b)
### Chart: Evenness index
| Category | Qiagen | | Zymo |
|---|---|---|---|
| Evenness | 0.6182388726662427 | None | 0.7022847783848334 |c)
