# Supplementary material for: Effects of DNA preservation solution and DNA extraction methods on microbial community profiling of soil
Source: Folia Microbiol (Praha). 2021 Apr 9;66(4):597–606. doi: 10.1007/s12223-021-00866-0 (PMC8298342; doi:10.1007/s12223-021-00866-0)
Supplement: Supplementary file 3 — Supplementary file3 Supplementary Figure 2 Alpha-diversity indices. DNA was extracted using two DNA extraction kits DNeasy PowerSoil Kit (Qiagen) and ZymoBIOMICS™ (Zymo). Standard deviations were calculated from two biological replicates. a) Richness represents the total number of OTUs per sample. b) Shannon represents the relative abundance of each OTU. c) Pielou’s evenness represents the evenness of OTUs abundances within the community profile. (DOCX 17 KB) [file 12223_2021_866_MOESM3_ESM.docx]

Supplementary Table 1. 16S rRNA gene primers used for Illumina MiSeq sequencing (Kozich et al. 2013).

| Primer | Sequence |
| --- | --- |
| V3F | CCTACGGGNGGCWGCAG |
| V4R | GGACTACHVGGGTWTCTAAT |

Supplementary Table 2 Relative abundances of the genera with a contribution of more than 10% in at least one soil sample. These data show the effect of the DNA preservation solution on the soil microbial profiles after 24 and 110 days of landfarming. DNA was extracted using the PowerSoil Kit (Qiagen) of soils from four experimental conditions: only fertilizer no-till, rhamnolipid 0.5g/kg and fertilizer, 5% w/w kenaf and, fertilizer and soil only (control). Numbers indicate the number of days of landfarming. Samples of 110 days of landfarming were additionally treated with a DNA preservation solution (+PS). Columns of 24 and 110+PS are averages of two duplicates. G+, Gram-positive; G-, Gram-negative.

|  | No till | | | Rhamnolipid | | | Kenaf | | | Soil only | | |
| --- | --- | --- | --- | --- | --- | --- | --- | --- | --- | --- | --- | --- |
| Taxa | 24 | 110 | 110+PS | 24 | 110 | 110+PS | 24 | 110 | 110+PS | 24 | 110 | 110+PS |
| *Bacillaceae* | 0.8 | 2.0 | 11.6 | 0.8 | 1.4 | 4.4 | 0.6 | 0.6 | 1.1 | 5.9 | 26.8 | 37.3 |
| *Bacillus* | 1.2 | 2.5 | 5.1 | 1.3 | 2.4 | 3.5 | 0.5 | 1.4 | 18.6 | 7.6 | 7.3 | 22.9 |
| *Lysinibacillus* | 0.2 | 0.5 | 2.6 | 0.2 | 0.4 | 6.4 | 0.1 | 0.2 | 12.1 | 1.4 | 1.3 | 6.4 |
| *Planococcaceae* | 0.0 | 0.1 | 8.5 | 0.0 | 0.0 | 0.6 | 0.0 | 0.0 | 0.2 | 0.2 | 0.2 | 0.6 |
| *Paenibacillus* | 0.1 | 0.2 | 10.9 | 0.1 | 0.1 | 7.5 | 0.0 | 0.1 | 23.6 | 0.4 | 0.4 | 17.6 |
| *Parvibaculum* | 9.8 | 5.5 | 2.0 | 9.7 | 3.1 | 1.1 | 6.0 | 1.5 | 0.7 | 0.0 | 0.0 | 0.0 |
| *Sphingomonas* | 0.3 | 1.6 | 0.5 | 0.4 | 2.5 | 2.4 | 1.2 | 19.8 | 2.2 | 6.9 | 6.5 | 1.1 |
| *Burkholderia* | 6.2 | 19.9 | 4.0 | 7.0 | 18.3 | 2.3 | 9.2 | 20.8 | 2.7 | 0.2 | 0.1 | 0.0 |
| *Enterobacter* | 0.0 | 0.0 | 11.7 | 0.0 | 0.0 | 9.6 | 0.2 | 0.0 | 6.8 | 0.0 | 0.0 | 0.0 |
| *Enterobacteriaceae* | 0.0 | 0.0 | 0.0 | 0.0 | 0.0 | 29.2 | 0.0 | 0.0 | 0.0 | 0.0 | 0.0 | 0.0 |
| *Immundisolibacter* | 21.7 | 3.9 | 1.0 | 16.7 | 4.6 | 1.0 | 15.7 | 0.3 | 0.5 | 0.2 | 0.0 | 0.0 |
| *Porticoccaceae;C1-B045* | 7.9 | 12.4 | 5.4 | 5.0 | 14.4 | 6.3 | 5.9 | 0.4 | 2.2 | 0.0 | 0.0 | 0.0 |
| *Pseudomonas* | 0.0 | 0.0 | 9.2 | 3.4 | 0.1 | 2.9 | 0.1 | 0.1 | 0.0 | 0.0 | 0.0 | 0.0 |
| *Rhodanobacteraceae* | 12.4 | 5.5 | 1.2 | 15.7 | 7.8 | 0.7 | 11.6 | 1.8 | 1.0 | 0.5 | 0.1 | 0.0 |
